# Association of Perceived Acoustic Environment Exposure and Nonrestorative Sleep with Cognitive Functioning Among Chinese Adults: A Cross-Sectional Survey

**DOI:** 10.3390/ijerph22050788

**Published:** 2025-05-16

**Authors:** Krista Ching Wai Chung, Sujin Nam, Jojo Yan Yan Kwok, Naomi Takemura, Hugh Davies, Lixi Huang, Daniel Yee Tak Fong

**Affiliations:** 1 School of Nursing, Li Ka Shing Faculty of Medicine, The University of Hong Kong, Hong Kong 999077, China; kristachung@connect.hku.hk (K.C.W.C.); jojoyyk@hku.hk (J.Y.Y.K.); 2 College of Nursing, Yonsei University, Seoul 03722, Republic of Korea; sujinnam1@yonsei.ac.kr; 3 School of Nursing, The Hong Kong Polytechnic University, Hong Kong 999077, China; naomi.takemura@polyu.edu.hk; 4 School of Population and Public Health, University of British Columbia, Vancouver, BC V6T 1Z3, Canada; hugh.davies@ubc.ca; 5 Department of Mechanical Engineering, The University of Hong Kong, Hong Kong 999077, China; lixi@hku.hk

**Keywords:** cognition, nonrestorative sleep, perceived acoustic environment, associated factors, moderation

## Abstract

Despite the growing emphasis on cognitive health, evidence regarding individual and environmental factors influencing cognitive functioning remains limited. We aimed to examine the association of personal and environmental factors, specifically perceived acoustic environment exposure and nonrestorative sleep, with cognitive functioning among Chinese adults. Between July and August 2022, we recruited 970 Chinese adults from all districts in Hong Kong for our cross-sectional study. Univariable and structured multiphase linear regression analyses were conducted to identify the contributory factors. Among 970 Chinese adults, the structured multiphase linear regression model revealed that being in their 30s (β = 0.160; 95% CI: 0.004, 0.315) and 40s (β = 0.186; 95% CI: 0.029, 0.343), being female (β = 0.095; 95% CI: 0.018, 0.173), being former smokers, and having medical consultations, medical histories (eczema, hearing problems, and insomnia), perceived acoustic environment exposure (traffic and mechanical sounds (β = 0.011; 95% CI: 0.008, 0.013), nature and music sounds (β = 0.004; 95% CI: 0.001, 0.007), and human sounds (β = 0.002; 95% CI: 0.0004, 0.005)), and psychological symptoms (perceived stress, depressive, and anxiety symptoms) were associated with worse cognitive functioning. Moreover, more nonrestorative sleep (β = −0.015; 95% CI: −0.022, −0.007) was also associated with worse cognitive functioning. This study revealed that increased perceived acoustic environment exposure and a higher degree of nonrestorative sleep were associated with poorer cognitive functioning among Chinese adults. This underscores the need for public health strategies and policies aimed at fostering a healthy acoustic environment and promoting sleep hygiene education in the community.

## 1. Introduction

Cognitive functioning encompasses a broad range of mental abilities, including learning, thinking, reasoning, remembering, problem-solving, decision-making, and attention [1]. While mild cognitive impairment may involve a moderate decline in one or more of these cognitive domains, with preserved daily life independence, dementia is diagnosed when such decline exacerbates to the extent that it interferes with everyday functional abilities [2]. Dementia represents a significant global health burden, with approximately 10 million new cases reported annually [3] and projections indicating a worldwide increase from 57.4 million cases in 2019 to 152.8 million in 2050 [4].

To promote healthy cognitive development, identify individuals at risk for cognitive decline, and develop effective interventions to preserve and enhance cognitive functioning, it is crucial to examine the determinants of cognitive functioning, including both individual and environmental factors. Individual factors affecting cognitive functioning encompass a wide range of aspects, including demographic, genetic, disease- and lifestyle-related, and social aspects [5]. Psychological distress has also been reported to adversely affect cognitive functioning [6]. Environmentally, living in densely populated cities has been associated with enhanced cognitive function [5], while higher levels of neighborhood economic disadvantage and exposure to environmental noise have been linked to poorer cognitive outcomes [7,8].

Nonrestorative sleep, characterized by an unrefreshed feeling upon awakening despite adequate sleep duration [9], has been associated with cognitive difficulties [10,11]. However, existing studies have limitations, such as using a single question from a non-validated questionnaire for assessing cognitive function or focusing on a narrow age range between 20 and 45 [11]. These limitations inform the need for further evaluation across broader demographics.

Excessive noise exposure, characterized as pervasive and unwanted sounds, has been linked to numerous health issues, including auditory dysfunction, cardiovascular diseases, mental illnesses, and cognitive impairment [12]. In Hong Kong, over 680,000 people are affected by noise pollution [13], exacerbated by limited habitable land resources leading to residential buildings near busy roads [14]. Cognitive reserve, which enhances resilience against cognitive decline [15], may be compromised by noise exposure, impairing cognitive functioning. Numerous studies have shown that everyday noises such as road traffic, music, and human conversations can detrimentally affect cognitive functions. Specifically, long-term road traffic noise exposure has been found to adversely affect executive functioning in older individuals [16]. Another study employing electroencephalography has revealed that certain noise types, particularly music and dialogue, negatively influence cognitive functions, with attention being particularly susceptible [17]. Moderate levels of mechanical noise have also demonstrated a significant impact on attention [17]. While a previous systematic review and meta-analysis suggested a relationship between environmental noise exposure and cognitive functioning, the evidence remains limited, particularly among older teenagers and young to middle-aged adults [8]. Furthermore, there is a scarcity of studies investigating self-reported exposure to the acoustic environment and its potential associations with cognitive functioning in the general population [18].

Consequently, our study aimed to examine the association of personal and environmental factors, specifically nonrestorative sleep and perceived acoustic environment exposure, with cognitive functioning among Chinese adults in Hong Kong. Additionally, we investigated whether the associations between cognitive functioning and several factors, including perceived acoustic environment exposure, psychological symptoms, and nonrestorative sleep, are moderated by age.

## 2. Materials and Methods

### 2.1. Study Design and Participant Recruitment

A cross-sectional study was conducted between July and August 2022, utilizing online questionnaires administered to the general population in Hong Kong. This study adhered to the Strengthening the Reporting of Observational Studies in Epidemiology (STROBE) guidelines (Table S1) [19]. Participants were recruited via a double-opt-in online panel service, receiving invitations through emails and text messages. Eligibility criteria included being (a) a Hong Kong resident, (b) aged 18 years or above, and (c) literate in traditional Chinese. The sample size was determined based on the guideline of 20 observations per independent variable for regression analysis as a recommended threshold to mitigate overfitting and ensure model stability [20]. We hypothesized that socio-demographic characteristics, medical consultations and medical histories, lifestyle behaviors, perceived acoustic environment exposure, psychological symptoms, and nonrestorative sleep would be associated with worse cognitive functioning. Specifically, we considered socio-demographic characteristics (19 variables), medical consultations and medical histories (8 variables), and lifestyle behaviors (6 variables) as the independent variables for our regression analysis. With these hypothesized factors summing to a total of 33 independent variables, a sample of 660 was required. Accounting for around 30% dropout, a target of 1000 participants was set. All participants provided informed consent online before commencing the survey.

### 2.2. Measurements

#### 2.2.1. Assessment of Perceived Cognitive Functioning

The 18-item Chinese version of the Cognitive Functioning Self-Assessment Scale (CFSS) was used to assess cognitive functioning [21]. Respondents were asked to rate the frequency of everyday life activities reflecting poor cognitive functioning within the past 12 months on a five-point Likert scale (1, indicating “never”, to 5, indicating “always”) [21]. The total score, ranging from 1 to 5, indicated cognitive function, with a higher score reflecting poorer cognitive functioning [21]. The Chinese version of the CFSS has been validated for construct validity and convergent validity, demonstrating high internal consistency with a Cronbach’s alpha coefficient of 0.94 and a McDonald’s omega hierarchical coefficient of 0.84 [21].

#### 2.2.2. Assessment of Perceived Acoustic Environment Exposure

Perceived acoustic environment exposure was assessed using a modified version of the Perceived Acoustic Environment (PAE) scale [22]. The original six-item English version comprised six items: traffic sounds, mechanical sounds from outside, nature sounds from outside, mechanical sounds within the dwelling, music within the dwelling, and sounds from human beings within the dwelling [22]. Each item asked for the frequency of exposure on a 5-point Likert scale (0, representing “never”, to 4, representing “all the time”) to a sound type at home in the past two weeks, and the corresponding pleasantness on a 5-point Likert scale (1, representing “very unpleasant”, to 5, representing “very pleasant”) [22]. The scale was translated into traditional Chinese independently by two bilingual registered nurses. The two Chinese versions were then reviewed by a panel of three members, including the two translators and a researcher experienced in transcultural adaptation. The panel obtained a consensus version, which was then back-translated by a third translator who was unaware of the original English version, and refinements were made where necessary. The scale then underwent cognitive debriefing with five individuals.

To enable the assessment of sound exposure outside the dwelling on a 5-point Likert scale (0, representing “never”, to 4, representing “all the time”), the panel included five additional items for outdoor environments and indoor workplaces/schools to address the exposure to mechanical sounds, music, and sounds from human beings. We also added an item on exposure to sounds from radio, television, or other video shows to enhance the scope of sources of sound exposure. Our subsequent exploratory factor analysis identified three subscales that were scored on a standardized scale of 0–100, using the following formula: (subscale score − minimum)/(maximum − minimum) × 100. A higher score indicates more frequent exposure in the past two weeks.

#### 2.2.3. Assessment of Perceived Stress

The 10-item Chinese version of the Perceived Stress Scale (PSS-10) was used to assess an individual’s perception of life as “overwhelming”, “uncontrollable”, and “unpredictable” [23]. Respondents rated the frequency of experiencing each statement over the past month on a five-point Likert scale (0, representing “never”, to 4, representing “very often”). The total score ranged from 0 to 40, with a higher total score indicating a higher perceived stress level. The Chinese version of the PSS-10 has been validated in a Chinese population with a Cronbach’s alpha coefficient of 0.70 [23].

#### 2.2.4. Assessment of Depressive Symptoms

The Chinese version of the Patient Health Questionnaire-9 (PHQ-9) is a self-administered 9-item instrument that assesses the presence and severity of depressive symptoms in an individual over the past two weeks [24]. Each item was scored from 0 to 3 (0 indicating “not at all”, 3 indicating “nearly every day”), with a total score ranging from 0 to 27. The Chinese version of the PHQ-9 has been validated in the general population of China with a Cronbach’s alpha coefficient of 0.82 [24].

#### 2.2.5. Assessment of Anxiety Symptoms

The Chinese version of the Generalized Anxiety Disorder-7 (GAD-7) is a self-reported 7-item instrument that assesses the frequency and severity of anxiety symptoms in the past two weeks [25]. Each item was scored as 0 (not at all), 1 (several days), 2 (over half the days), or 3 (nearly every day), with a total score ranging from 0 to 21. The Chinese version of the GAD-7 has been validated among Chinese outpatients with epilepsy, demonstrating good internal consistency with a Cronbach’s alpha coefficient of 0.89 [25].

#### 2.2.6. Assessment of Nonrestorative Sleep

Nonrestorative sleep was assessed using the 9-item Chinese version of the Nonrestorative Sleep Scale (NRSS), which evaluates nonrestorative sleep over the past month [26]. Each item was rated on a scale ranging from 1 (very poor) to 10 (very good). The original score for each item was converted to a weighted score between 1 and 5. Subsequently, specific items with negative wordings, namely, items 4, 5, and 9, were reversed. The total score ranges from 9 to 45, with a higher score indicating less nonrestorative sleep. The Chinese version of the NRSS has been validated with a Cronbach’s alpha value of 0.84 [26].

#### 2.2.7. Assessment of Socio-Demographic, Medical, and Lifestyle Characteristics

Socio-demographic information, including age (18–29, 30–39, 40–49, 50–59, or 60 or above), gender (male or female), educational attainment (with or without a university degree), employment status (non-working or working), monthly household income (<HKD 5000–29,999, HKD 30,000–49,999, or >HKD 50,000), and housing type (public rental housing unit; subsidized-sale flat; private residential flat; modern village house, villa, or bungalow; or other) were collected.

The number of medical consultations in the past year was assessed by a self-developed item: “How many times did you visit the doctor in the past year?”. Participants also reported their medical histories, including eczema, hyperlipidemia, diabetes mellitus, hearing problems, cardiovascular diseases, irritable bowel syndrome, gastric ulcers, and insomnia, with response options of “yes” or “no”.

Furthermore, lifestyle behaviors such as smoking and drinking patterns were evaluated using self-developed items. Participants were asked, “Do you smoke or have you quit smoking?” and “Do you drink alcohol or have you abstained from drinking?”, with three response options provided for each behavior: “never”, “former smoker/drinker”, or “current smoker/drinker”.

### 2.3. Statistical Analyses

All statistical analyses were conducted using R (version 4.2.1). To assess the psychometric performance of the modified PAE, we conducted an exploratory factor analysis (EFA) using the “psych” package. Sample adequacy was evaluated through a significant Bartlett’s test of sphericity and a Kaiser–Meyer–Olkin (KMO) value exceeding 0.50 [27]. The number of factors was determined by parallel analysis [27]. Maximum likelihood estimation with oblimin rotation was used to estimate the factor loadings, with factor loadings greater than 0.30 required for inclusion [27]. Internal consistency was evaluated using Cronbach’s alpha coefficient, with values above 0.70 considered acceptable [27].

To identify determinants of cognitive functioning, we first conducted univariable regression analyses of the CFSS score on personal and environmental factors. Subsequently, a structured multiphase regression was conducted to assess these factors after adjusting for potential confounders [28]. Specifically, we grouped the factors into six clusters: Cluster 1 as socio-demographic characteristics, Cluster 2 as medical consultations and medical histories, Cluster 3 as lifestyle behaviors, Cluster 4 as perceived acoustic environment exposure, Cluster 5 as psychological symptoms, and Cluster 6 as nonrestorative sleep. The grouping was made with the hypothesis that the variables in Cluster 1 causally affect those in Clusters 2, 3, 4, 5, and 6, but not the reverse; the variables in Cluster 2 may influence variables in subsequent clusters, but not vice versa; etc. Then, the variables were entered sequentially into the regression model in phases. In Phase 1, only the variables in Cluster 1 were entered, not variables in subsequent clusters, to avoid over-adjustment. In Phase 2, the variables in Cluster 2 were entered with adjustment of the variables in Cluster 1. Again, no variables in subsequent clusters were entered to avoid over-adjustment. In Phase 3, the variables in Cluster 3 were entered with adjustment of the variables in Clusters 1 and 2, and so on. The effect of a variable was taken in the phase in which the variable was entered, not for the purpose of adjustment. This avoided potential over-adjustment by refraining from adjusting variables from later phases in which the variable was hypothesized as a causal factor [28]. Moreover, to explore any moderating effect of age, its interaction with perceived acoustic environment exposure, psychological symptoms, and nonrestorative sleep were examined in Phases 4, 5, and 6, respectively. The presence of multicollinearity was assessed using the variance inflation factor (VIF) obtained by the “car” package. Multicollinearity was deemed present when it showed a value of 10 or above [20]. Finally, model adequacy was evaluated by analyzing the normal probability plot of the standardized residuals and the scatter plot of the standardized residuals against the predicted values. All significance tests used 5% as the nominal level of significance.

## 3. Results

### 3.1. Sample Characteristics

Among 1002 enrolled participants, 32 cases were excluded due to inconsistent demographic information. The remaining 970 participants were included in our data analysis (Table 1). The mean age of the sample was 40 (SD = 14) years, ranging from 18 to 81 years. Females constituted 56.2% of the sample. Eczema was the most frequently reported medical history, with a prevalence of 15.1%. Over 20% of the participants were former or current smokers, whereas more than half reported being former or current drinkers. The mean scores for the CFSS, PSS, PHQ-9, GAD-7, and NRSS were 2.5 (SD = 0.6), 18.2 (SD = 4.9), 6.2 (SD = 5.4), 5.9 (SD = 4.5), and 31.0 (SD = 5.5), respectively.

### 3.2. Factor Structure of the Modified PAE

Table 2 presents the results of the EFA of the modified PAE (*n* = 970). The data demonstrated sample adequacy, as confirmed by a significant Bartlett’s test of sphericity (Χ^2^ = 3750.34, degrees of freedom (df) = 66, *p* < 0.001) and a KMO value of 0.87, which surpassed the recommended value of 0.50. The modified PAE yielded a three-factor structure based on parallel analysis, explaining 48% of the total variance. All items exhibited factor loadings exceeding the minimum value of 0.30. For the first factor, “traffic and mechanical sounds”, factor loadings ranged between 0.51 and 0.83. For the second factor, “nature and music sounds”, factor loadings ranged from 0.40 to 0.74. For the third factor, “human sounds”, factor loadings ranged from 0.35 to 0.81. The Cronbach’s alpha coefficients for the whole scale and the first, second, and third factors were 0.84, 0.78, 0.74, and 0.77, respectively, all exceeding the acceptable value of 0.70. The standardized sub-total exposure frequency scores for (a) traffic and mechanical sounds, (b) nature and music sounds, and (c) human sounds were 27.7 (SD = 15.9), 25.0 (SD = 15.5), and 39.2 (SD = 18.6), respectively.

### 3.3. Factors Associated with Cognitive Functioning

Table 3 presents the results of a univariable regression, examining the association between various factors and cognitive functioning. Specifically, age groups, including the 18–29 (β = 0.169; 95% CI: 0.024, 0.313), 30–39 (β = 0.171; 95% CI: 0.021, 0.322), and 50–59 groups (β = 0.188; 95% CI: 0.036, 0.341); gender (β = 0.104; 95% CI: 0.027, 0.181); number of medical consultations (β = 0.029; 95% CI: 0.019, 0.039); eczema (β = 0.221; 95% CI: 0.115, 0.328); hearing problems (β = 0.362; 95% CI: 0.146, 0.579); cardiovascular diseases (β = 0.273; 95% CI: 0.036, 0.509); irritable bowel syndrome (β = 0.367; 95% CI: 0.121, 0.613); gastric ulcers (β = 0.294; 95% CI: 0.025, 0.564); insomnia (β = 0.403; 95% CI: 0.127, 0.678); history of smoking (β = 0.252; 95% CI: 0.134, 0.371); alcohol drinking, including both former drinkers (β = 0.140; 95% CI: 0.004, 0.276) and current drinkers (β = 0.085; 95% CI: 0.005, 0.165); perceived acoustic environment exposure, including traffic and mechanical sounds (β = 0.015; 95% CI: 0.013, 0.018), nature and music sounds (β = 0.012; 95% CI: 0.010, 0.015), and human sounds (β = 0.008; 95% CI: 0.006, 0.010); psychological symptoms, including symptoms of stress (β = 0.060; 95% CI: 0.053, 0.067), depression (β = 0.060; 95% CI: 0.054, 0.066), and anxiety (β = 0.070; 95% CI: 0.063, 0.078); and nonrestorative sleep (β = −0.049; 95% CI: −0.055, −0.042) were found to be statistically significant in their associations with cognitive functioning.

Table 4 displays the results of a structured multiphase regression analysis examining the effects of various factors on cognitive functioning. The VIFs ranged between 1.01 and 1.79, indicating no multicollinearity. Participants aged 30–39 and 40–49 reported higher CFSS scores than those aged 60 or above. Additionally, being female; having a higher frequency of medical consultations; experiencing eczema, hearing problems, and insomnia; and having a history of smoking were significantly associated with higher CFSS scores. Among all types of perceived acoustic environment, exposure to traffic and mechanical sounds had the largest negative impact on the CFSS score (β = 0.011; 95% CI: 0.008, 0.013). Furthermore, perceived stress, depressive, and anxiety symptoms were all significantly associated with worse cognitive functioning (Table 4). In contrast, less nonrestorative sleep was associated with better cognitive functioning (β = −0.015; 95% CI: −0.022, −0.007). No model inadequacies were revealed after checking model residuals.

Furthermore, our interaction analysis showed a significant interaction between age and anxiety symptoms on cognitive functioning (β = −0.001, 95% CI: −0.002, −0.0002). However, our results did not indicate significant interactions between age and perceived acoustic environment exposure on cognitive functioning (*p* > 0.085). Age also did not moderate the effects of perceived stress, depressive symptoms, and nonrestorative sleep on CFSS (*p* > 0.190).

## 4. Discussion

This study is the first to comprehensively investigate the association of perceived sound exposure and nonrestorative sleep with cognitive functioning using standardized scales and adjusting for related confounders. More frequent perceived acoustic environment exposure, nonrestorative sleep, and psychological symptoms were all associated with poorer cognitive functioning. By including a wide age range of participants from 18 to 81 years, our study addressed the significant need for research involving older teenagers and young to middle-aged adults to assess these associations. Also, we found a moderating effect of age on the association between anxiety symptoms and cognitive functioning, but not on the associations of perceived acoustic environment exposure and nonrestorative sleep with cognitive functioning.

Our results indicate that all types of sound exposure are generally associated with impaired cognitive functioning. Noise may impair cognitive functioning through its effects on attention and cognitive load. Unlike previous studies that relied on estimated noise exposure levels [29] or controlled experimental settings [30], our study captured the real-world living environments of individuals. According to the load theory of selective attention and cognitive control [31], exposure to noise forces the brain to allocate additional perceptual capacity to filter out irrelevant auditory stimuli, which can disrupt the encoding and consolidation of information, thus impairing memory retention and recall.

All three types of noise exposure, including traffic and mechanical sounds, nature and music sounds, and human sounds, showed detrimental effects on cognitive functioning. The effects may be due to their often low sound frequencies [18]. The vibration aspect of such low-frequency noise (LFN) may negatively impair our circulatory, endocrine, and nervous systems, as well as our learning [18]. Nevertheless, while traffic and mechanical sounds have been well documented to impair cognitive functions, it is noteworthy that, in our study, natural sounds also had detrimental impacts on cognitive functioning, which contrasts with two previous studies that showed a positive association of nature sounds with cognitive restoration [32,33]. Both studies utilized pre-recorded nature sounds, including birds, rainfall, waves, wind, and others. In one study, participants aged 19–44 years received these sounds for 15 min once [32], while the other study involved university students who listened to nature sounds for 30 min each day over four weeks [33]. The positive association observed in these studies may be attributed to the controlled sound exposure, which masked environmental noise. The effect is particularly plausible in the study involving university students, as they listened to nature sounds through earphones. Conversely, our study evaluated sound exposure in everyday life, where sounds are encountered unintentionally and without control of their intensity level. Additionally, in an urbanized city like Hong Kong, exposure to nature sounds, such as wind, rain, and thunder, can often be disruptive without the presence of birds and insects, which might serve as distractors when performing cognitive tasks. Music sounds in our study also showed a detrimental association with cognitive functioning, corroborating previous research which has shown that irrelevant music can impair performance on recall tasks [34,35] due to distraction or emotional arousal and valence.

Among the three types of perceived acoustic environment exposure, human sounds had the least detrimental impact on cognitive functioning despite having the highest average exposure. This could be attributed to habituation and adaptive tolerance developed over time due to prolonged exposure to common human sounds. In highly urbanized areas like Hong Kong, growing public awareness and concern regarding environmental soundscape management may lead to a greater desire for quieter environments. As residents can freely choose and prioritize quieter environments, they may become more sensitive to environmental noise that was previously tolerated, even if exposure levels remain unchanged. This heightened sensitivity could result in more frequent noise complaints. Therefore, it is worth investigating whether the growing societal emphasis on soundscape quality could paradoxically reduce noise tolerance as more individuals seek quieter sound environments. Moreover, we did not differentiate between the different types of human sounds, such as laughter, footsteps, and children at play. Future research could investigate the effects of exposure to various types of human sounds other than irrelevant speech sounds on cognitive performance among community-dwelling adults at indoor workplace settings.

The association of nonrestorative sleep with impaired cognitive functioning across a broad age range from 18 to 81 years among the Chinese population has not been reported before. Earlier research was limited to young United States (U.S.) adults (aged 20–45) [11] and Finnish older adults in their 60s [36], both of which focused on executive functioning [11,36]. Conversely, our study evaluated perceived cognitive functioning, encompassing a broader spectrum of domains, including spatio-temporal orientation, attention, and memory. Nonrestorative sleep and sleep quality/quantity are distinct constructs such that one may still feel not restored even after adequate sleep [27,37]. Our finding, aligning with previous research, reinforces the notion that the restorative quality of sleep, rather than just sleep quantity and quality, is critical for optimal cognitive abilities. Although the underlying mechanisms remain unclear, some studies have suggested a link between nonrestorative sleep and metabolic syndrome [38], which in turn may impair cognitive functioning [39]. Further studies would be needed to determine the underlying mechanism across the lifespan.

Our study also revealed that age moderated the association between anxiety symptoms and cognitive functioning. Contrary to a previous Scottish study involving 286 adults which reported that the detrimental impact of anxiety on cognitive health may exacerbate with increasing age [40], our findings suggest that anxiety exerts a less detrimental effect on cognitive functioning as Chinese individuals grow older. While both studies encompassed a broad age range, our study included a larger sample size, enabling a more nuanced assessment of age-specific influences. In contrast, the Scottish study was limited to categorizing participants into young, middle-aged, and older age groups. Additionally, the observed difference may also be attributed to the higher emotion regulation strategies shaped by collectivist values in Chinese society [41], enabling older Chinese adults in our study to better cultivate adaptive strategies to buffer the impact of anxiety-related cognitive decline. While the underpinning neural mechanisms remain uncertain, future studies are warranted for a thorough and definite understanding of the interplay between age, anxiety, and cognitive functioning for promoting cognitive health across age groups.

Contrasting with prior studies that showed age-varying effects of depression on cognitive functioning among U.S. immigrants aged 60 to 80 [42], our study found no moderating role of age for depression on cognitive functioning among Chinese adults aged 18 to 81. This divergence may be due to methodological differences across studies, as our inclusion of a broader age range encompassing younger and healthier adults might have diluted age-related effects, particularly as the impact of depression on cognition may manifest progressively in later life [43]. Conversely, our finding aligns with a U.S. study of adults aged 45 or above [44], which also found no age moderation in the relationship between stress and cognitive functioning. This consistency in findings may reflect that the influence of stress on cognitive functioning is less susceptible to aging across cultures. Future longitudinal studies are needed to verify any temporal patterns in how mental stressors could influence cognitive health across the lifespan.

Our study results carry significant implications for diverse stakeholders. For the general population, our findings highlight the need for minimizing daily exposure to disruptive sounds (including traffic and mechanical sounds, natural and music sounds, and human sounds) in everyday life, particularly during cognitive tasks. For individuals who feel unrefreshed after sleep despite having adequate sleep duration, this may signify a need for addressing sleep quality through behavioral modifications or professional consultations. For policymakers, sound mitigation could be prioritized for sounds with the strongest cognitive decline risks. Furthermore, public health campaigns not only can promote hearing health but also help increase awareness of nonrestorative sleep as a detrimental factor for cognitive decline and advocate for sleep hygiene education. For researchers, future studies could incorporate longitudinal designs with objective measurements to disentangle our observed associations between perceived noise exposure, nonrestorative sleep, and cognitive decline. Moreover, the mechanisms behind the unexpected detrimental impact of natural and music sounds and human sounds are worth exploring, and a finer classification of these sounds may be beneficial for future studies.

Several limitations are worth noting. First, the use of self-reported data collected through an online survey platform might have introduced inconsistencies and recall biases. To mitigate these risks, we implemented validity-check questions at random places within the questionnaire. Second, our data collection by online questionnaires may have constrained our ability to reach individuals with limited internet access, low socio-economic status, and insufficient digital literacy. To address this inherent selection bias, future studies should consider integrating offline data collection to improve the generalizability of findings. Third, the cross-sectional study design limited the ability to establish causal inference and precluded the determination of the directionality of the observed associations between determinants and cognitive functioning. For instance, while the relationship between perceived noise exposure and cognitive functioning could suggest that chronic noise exposure leads to cognitive decline, it is also plausible that pre-existing cognitive decline may heighten noise sensitivity and thus more frequent episodes of perceived noise exposure. Future longitudinal studies are needed to examine the long-term effects of determinants, particularly perceived acoustic environment exposure and nonrestorative sleep, on cognitive functioning. Given that cognitive decline tends to build up progressively, longitudinal follow-up of the same cohort across multiple timepoints is necessary to better capture the dynamic interactions and minimize survivor bias. Lastly, relying on self-reported scales to evaluate acoustic environment exposure might be susceptible to response bias. Nonetheless, we made efforts to minimize bias by using a modified version of the PAE with appropriate translation, transcultural adaptation, and psychometric validation.

## 5. Conclusions

This study highlights the importance of perceived acoustic environment exposure and nonrestorative sleep in influencing cognitive functioning among Chinese adults. By recognizing the role of environmental and individual factors in cognitive health, policymakers can devise strategies to cultivate a healthier acoustic environment and promote better sleep hygiene. We recommend raising public awareness about the detrimental impacts of environmental acoustic stressors and nonrestorative sleep on cognitive health. This could be achieved through educational campaigns that emphasize the importance of protecting hearing, minimizing exposure to harmful sounds, and maintaining good sleep hygiene. Such initiatives would promote cognitive well-being across the community.

## Figures and Tables

**Table 1:** Characteristics of survey participants (*n* = 970); Association of perceived acoustic environment exposure and nonrestorative sleep with cognitive functioning among Chinese adults: a cross-sectional survey, Chung, 2025.

Variables	Participants
Age (years), *n* (%)	
18–29	289 (29.8)
30–39	211 (21.8)
40–49	192 (19.8)
50–59	189 (19.5)
≥60	89 (9.2)
Gender, *n* (%)	
Male	425 (43.8)
Female	545 (56.2)
Education level, *n* (%)	
Without a university degree	306 (31.5)
University degree or above	664 (68.5)
Employment status, *n* (%)	
Working	875 (90.2)
Non-working	95 (9.8)
Monthly household income (HKD)	
<5000–29,999	238 (24.5)
30,000–49,999	377 (38.9)
≥50,000	355 (36.6)
Housing type, *n* (%)	
Public rental housing unit	219 (22.6)
Subsidized-sale flat	141 (14.5)
Private residential flat	560 (57.7)
Modern village house, villa, or bungalow	34 (3.5)
Other (e.g., staff quarters, student residence, or temporary quarters)	16 (1.6)
Medical consultations in the past year, mean (SD)	2.8 (3.8)
Medical histories, *n* (%)	
Eczema	146 (15.1)
Hyperlipidemia	95 (9.8)
Diabetes mellitus	34 (3.5)
Hearing problems	31 (3.2)
Cardiovascular diseases	26 (2.7)
Irritable bowel syndrome	24 (2.5)
Gastric ulcers	20 (2.1)
Insomnia	19 (2.0)
Smoking status, *n* (%)	
Never	742 (76.5)
Former	113 (11.6)
Current	115 (11.9)
Alcohol drinking, *n* (%)	
Never	443 (45.7)
Former	434 (44.7)
Current	93 (9.6)
Modified PAE (range: 0–100), mean (SD)	
Traffic and mechanical sounds	27.7 (15.9)
Nature and music sounds	25.0 (15.5)
Human sounds	39.2 (18.6)
CFSS (range: 1–5), mean (SD)	2.5 (0.6)
Spatio-temporal orientation (range: 1–5), mean (SD)	2.4 (0.7)
Attention (range: 1–5), mean (SD)	2.6 (0.7)
Memory (range: 1–5), mean (SD)	2.5 (0.7)
PSS (range: 0–40), mean (SD)	18.2 (4.9)
PHQ-9 (range: 0–27), mean (SD)	6.2 (5.4)
GAD-7 (range: 0–21), mean (SD)	5.9 (4.5)
NRSS (range: 9–45), mean (SD)	31.0 (5.5)

CFSS, Cognitive Functioning Self-Assessment Scale; GAD-7, Generalized Anxiety Disorder; Modified PAE, a modified version of the Perceived Acoustic Environment; NRSS, Nonrestorative Sleep Scale; PSS, Perceived Stress Scale; PHQ-9, Patient Health Questionnaire.

**Table 2:** The results of exploratory factor analysis (*n* = 970); Association of perceived acoustic environment exposure and nonrestorative sleep with cognitive functioning among Chinese adults: a cross-sectional survey, Chung, 2025.

Items	Exposure Frequency (Range: 0–4), Mean (SD)	Factor 1 (Traffic and Mechanical Sounds)	Factor 2 (Nature and Music Sounds)	Factor 3 (Human Sounds)	Cronbach’s Alpha	Total Cronbach’s Alpha
1. Traffic noise, e.g., cars, buses, airplanes, and trains	1.43 (0.87)	0.60			0.78	0.84
2. Mechanical noise: from outside, e.g., sirens, construction, and machines	1.08 (0.78)	0.83				
3. Mechanical noise: in dwelling, e.g., appliances, plumbing, and elevators	1.02 (0.81)	0.60				
4. Mechanical noise: school/indoor work area (not including work from home)	0.91 (0.81)	0.51				
5. Nature sounds from outside, e.g., singing birds, flowing water, wind blowing, and rustling leaves	1.08 (0.84)		0.40		0.74	
6. Music: outdoor	0.97 (0.83)		0.74			
7. Music: within the dwelling	1.12 (0.81)		0.64			
8. Music: school/indoor work area (not including work from home)	0.82 (0.83)		0.63			
9. Sounds from human beings (e.g., conversation, laughter, children at play, and footsteps) from outside	1.65 (1.00)			0.81	0.77	
10. Sounds from human beings (e.g., conversation, laughter, children at play, and footsteps) within the dwelling	1.49 (0.94)			0.80		
11. Sounds from human beings (e.g., conversation, laughter, children at play, and footsteps) at school/indoor work area (excluding work from home)	1.55 (1.01)			0.71		
12. Sound from TV/radio/media episodes	1.58 (0.92)			0.35		
Variance explained (%)		16.0%	15.0%	17.0%		
Total variance explained (%)		48.0%				

**Table 3:** Univariable regression of cognitive functioning among Chinese adults (*n* = 970); Association of perceived acoustic environment exposure and nonrestorative sleep with cognitive functioning among Chinese adults: a cross-sectional survey, Chung, 2025.

Variables	Estimate	95% CI	*p* Value
Age (reference: age 60 or above)			
18–29	0.169	(0.024, 0.313)	0.022
30–39	0.171	(0.021, 0.322)	0.026
40–49	0.188	(0.036, 0.341)	0.016
50–59	0.028	(−0.125, 0.181)	0.720
Gender (reference: male)			
Female	0.104	(0.027, 0.181)	0.008
Education level (reference: without a university degree)			
University degree or above	0.012	(−0.071, 0.095)	0.776
Employment status (reference: non-working)			
Working	0.067	(−0.062, 0.196)	0.309
Monthly household income (HKD) (reference: <5000−29,999)			
30,000–49,999	0.029	(−0.069, 0.128)	0.560
≥50,000	−0.032	(−0.132, 0.068)	0.533
Housing type (reference: public rental housing unit)			
Subsidized-sale flat	−0.068	(−0.197, 0.061)	0.302
Private residential flat	−0.055	(−0.151, 0.040)	0.254
Modern village house, villa, or bungalow	0.010	(−0.211, 0.230)	0.931
Other	−0.160	(−0.469, 0.150)	0.312
Medical consultations in the past year	0.029	(0.019, 0.039)	<0.001
Medical histories (reference: disease-free)			
Eczema	0.221	(0.115, 0.328)	<0.001
Hyperlipidemia	0.126	(−0.003, 0.255)	0.056
Diabetes mellitus	0.146	(−0.063, 0.354)	0.171
Hearing problems	0.362	(0.146, 0.579)	0.001
Cardiovascular diseases	0.273	(0.036, 0.509)	0.024
Irritable bowel syndrome	0.367	(0.121, 0.613)	0.003
Gastric ulcers	0.294	(0.025, 0.564)	0.032
Insomnia	0.403	(0.127, 0.678)	0.004
Smoking status (reference: never)			
Former	0.252	(0.134, 0.371)	<0.001
Current	−0.021	(−0.141, 0.098)	0.728
Alcohol drinking (reference: never)			
Former	0.140	(0.004, 0.276)	0.0434
Current	0.085	(0.005, 0.165)	0.0385
Perceived Acoustic Environment exposure (range: 0–100)			
Traffic and mechanical sounds	0.015	(0.013, 0.018)	<0.001
Nature and music sounds	0.012	(0.010, 0.015)	<0.001
Human sounds	0.008	(0.006, 0.010)	<0.001
Psychological symptoms			
Perceived stress (range: 0–40)	0.060	(0.053, 0.067)	<0.001
Depressive symptoms (range: 0–27)	0.060	(0.054, 0.066)	<0.001
Anxiety symptoms (range: 0–21)	0.070	(0.063, 0.078)	<0.001
Nonrestorative sleep (range: 9–45)	−0.049	(−0.055, −0.042)	<0.001

**Table 4:** Structured multiphase regression analysis of cognitive functioning among Chinese adults (*n* = 970); Association of perceived acoustic environment exposure and nonrestorative sleep with cognitive functioning among Chinese adults: a cross-sectional survey, Chung, 2025.

Variables	Estimate	95% CI	*p* Value
Phase 1: Socio-demographic characteristics (n = 970, R^2^ = 2.39%, adjusted R^2^ = 1.06%)			
Age (reference: age 60 or above)			
18–29	0.147	(−0.004, 0.297)	0.057
30–39	0.160	(0.004, 0.315)	0.045
40–49	0.186	(0.029, 0.343)	0.020
50–59	0.026	(−0.129, 0.180)	0.743
Gender (reference: male)			
Female	0.095	(0.018, 0.173)	0.016
Education level (reference: without a university degree)			
University degree or above	−0.017	(−0.105, 0.072)	0.711
Monthly household income (HKD) (reference: <5000 –29,999)			
30,000–49,999	0.022	(−0.081, 0.126)	0.671
≥50,000	−0.036	(−0.147, 0.075)	0.519
Employment status (reference: non-working)			
Working	0.063	(−0.073, 0.199)	0.367
Housing type (reference: public rental housing unit)			
Subsidized-sale flat	−0.051	(−0.183, 0.080)	0.446
Private residential flat	−0.033	(−0.136, 0.070)	0.533
Modern village house, villa, or bungalow	0.020	(−0.200, 0.240)	0.859
Other	−0.119	(−0.428, 0.191)	0.453
Phase 2: Medical consultations and histories (*n* = 970, R^2^ = 9.21%, adjusted R^2^ = 7.88%)			
Medical consultations	0.022	(0.012, 0.032)	<0.001
Medical histories (reference: disease-free)			
Eczema	0.173	(0.068, 0.278)	0.001
Hyperlipidemia	0.113	(−0.021, 0.246)	0.099
Diabetes mellitus	0.151	(−0.057, 0.360)	0.155
Hearing problems	0.368	(0.157, 0.579)	<0.001
Cardiovascular diseases	0.152	(−0.085, 0.390)	0.208
Irritable bowel syndrome	0.220	(−0.021,0.461)	0.074
Gastric ulcers	0.145	(−0.118, 0.408)	0.280
Insomnia	0.284	(0.013, 0.555)	0.040
Phase 3: Lifestyle behaviors (*n* = 970, R^2^ = 9.62%, adjusted R^2^ = 8.40%)			
Smoking status (reference: never)			
Former	0.220	(0.094, 0.345)	<0.001
Current	−0.045	(−0.166, 0.077)	0.472
Alcohol drinking (reference: never)			
Former	0.003	(−0.138, 0.145)	0.963
Current	0.062	(−0.020, 0.143)	0.138
Phase 4: Perceived acoustic environment exposure (*n* = 970, R^2^ = 23.04%, adjusted R^2^ = 21.91%)			
Traffic and mechanical sounds	0.011	(0.008, 0.013)	<0.001
Nature and music sounds	0.004	(0.001, 0.007)	0.004
Human sounds	0.002	(0.0004, 0.005)	0.021
Phase 5: Psychological symptoms (*n* = 970, R^2^ = 41.91%, adjusted R^2^ = 40.87%)			
Perceived stress	0.029	(0.021, 0.037)	<0.001
Depressive symptoms	0.023	(0.014, 0.032)	<0.001
Anxiety symptoms	0.017	(0.006, 0.028)	<0.001
Phase 6: Nonrestorative sleep (*n* = 970, R^2^ = 42.85%, adjusted R^2^ = 41.77%)			
Nonrestorative sleep	−0.015	(−0.022, −0.007)	<0.001

## Data Availability

Data may be made available upon reasonable request to the corresponding author.

## Supplementary Materials

The following supporting information can be downloaded at: https://www.mdpi.com/article/10.3390/ijerph22050788/s1, Table S1: STROBE Statement—Checklist of items that should be included in reports of cross-sectional studies.
